# Lysergic Acid Diethylamide, Psilocybin and Dimethyltryptamine in Depression Treatment: A Systematic Review

**DOI:** 10.3390/ph14080793

**Published:** 2021-08-12

**Authors:** Gniewko Więckiewicz, Iga Stokłosa, Magdalena Piegza, Piotr Gorczyca, Robert Pudlo

**Affiliations:** Department and Clinic of Psychiatry, Medical University of Silesia, 42-612 Tarnowskie Góry, Poland; iga.florczyk@gmail.com (I.S.); mpiegza@sum.edu.pl (M.P.); pgorczyca@sum.edu.pl (P.G.); rpudlo@sum.edu.pl (R.P.)

**Keywords:** lysergic acid diethylamide (LSD), psilocybin, dimethyltryptamine (DMT), depression

## Abstract

Despite many different kinds of substances available for depression treatment, depression itself still appears to be a clinical challenge. Recently, formerly illicit substances came to scientists’ attention, including lysergic acid diethylamide (LSD), psilocybin and dimethyltryptamine (DMT). Some studies suggest that these substances might be effective in depression treatment. The aim of this study was to evaluate the efficiency of LSD, psilocybin and DMT in depression treatment in the light of current medical literature. The authors followed the Preferred Reporting Items for Systematic Review and Meta-Analysis (PRISMA) guidelines for this systematic review. The authors searched the PubMed and Cochrane Library databases to identify relevant publications. Finally, 10 papers were included. Most of the selected studies showed significant correlation between psilocybin and DMT use and reduction in depression symptom intensity. By analyzing qualified studies, it can be concluded that psilocybin and DMT could be useful in depression treatment, but further observations are still required.

## 1. Introduction

Psychedelic substances have accompanied humanity around the world for millennia. Indigenous peoples of South America have used herbal dimethyltryptamine in folk medicine and religious practice for at least a thousand years, and the 6000-year-old Spanish pictographs probably show the local species of the hallucinogenic mushroom *Psilocybe hispanica* [1,2]. While psychodysleptic substances can be dangerous to the user when used inappropriately, they can provide benefits under clinical conditions, as evidenced by the increasing number of clinical trials on the use of psychedelics in treatment.

Lysergic acid diethylamide (LSD) is a semisynthetic substance derived from the lysergic acid of the fungus *Caliceps pupurea*. LSD is mainly taken orally, but it can also be smoked, snorted or injected [3]. This substance exists in the form of four isomers, of which only the d-LSD form has psychoactive properties [4]. LSD has been shown to be an agonist of 5HT2a, 5HT1a and 5HT2c receptors. By affecting the 5HT2a receptor (and indirectly by enhancing glutamatergic transmission in the prefrontal cortex and alterations in cortico-cortical and cortico-subcortical transmission), LSD use results in a hallucinogenic effect [5]. The effects of LSD use are also associated with its pleiotropic effects because, in addition to its affinity for serotonin receptors, LSD also affects dopamine receptors (D1, D2, D4) and indirectly affects glutamatergic neurotransmission and TAAR receptors (in animal models). LSD also affects alpha-2 adrenergic receptors to a small extent, stimulating the sympathetic nervous system, resulting in an increase in body temperature, sweating, tachycardia, increased blood pressure and muscle tension, which are the first to occur after LSD ingestion [6]. Doses greater than 100 ug cause heightened sensory perceptions, synesthesias, pseudohallucinations, changes in time perception, feelings of depersonalization and derealization [7]. Unlike other psychoactive substances, no physical dependence has been observed, and low toxicity has been noted [8].

Psilocybin is one of the major psychedelic agents found in certain species of mushrooms around the world. Psilocybin is an agonist of 5HT2a serotonin receptors; however, unlike LSD, it has no effect on dopamine receptors [9]. The undisputed advantage of this tryptamine derivative is its low toxicity, minimal side effects and lack of substance dependence [10]. The effects of psilocybin depend on the dose administered—at an amount of about 20 mg taken at one time, users experience a state of altered consciousness, increased introspection and hypnagogic experiences. Perceptual changes such as synesthesia, delusions and alterations in the sense of time are also observed. The effects of the hallucinogen are mainly associated with the activation of 5HT2a receptors in the thalamus, reducing the activity of these areas [9,11]. In animal models, vegetative changes such as mydriasis, tachycardia, a slight increase in blood pressure and hyperglycemia have also been observed after ingestion of this substance. The main metabolite of psilocybin, produced by hepatic metabolism, is psilocin, and the mean elimination time of it is about 50 min [11].

DMT, or dimethyltryptamine, is a psychedelic substance commonly found in plants and in the organisms of some mammals, including humans. DMT is produced endogenously in the pineal gland in small amounts, and its role is not yet known [12]. DMT is an agonist of 5-HT1A, 5-HT1B, 5-HT1D, 5-HT1E, 5-HT2A, 5-HT2B and 5-HT2C receptors and a partial agonist of 5-HT5A, 5-HT6 and 5-HT7. The hallucinogenic effect is mainly achieved by stimulating the 5-HT2A receptor and enhancing presynaptic glutamatergic transmission in the prefrontal cortex [13]. DMT can be ingested, inhaled, injected intravenously or inflamed. In the first case, it must be taken together with monoamine oxidase A inhibitors since DMT is metabolized in the first pass through the liver with the help of this enzyme [14]. Depending on the route of ingestion, DMT causes psychedelic effects of varying severity (from mild agitation to visual and auditory hallucinations) depending on the metabolite content in plasma, but the subjective effects remain similar to those associated with the use of LSD and psilocybin, which is associated with similar effects of these substances via the 5-HT2A receptor to the central nervous system [15,16]. The most common side effects of DMT use are vomiting and diarrhea [13]. It is estimated that ingestion of DMT at doses used to achieve psychedelic effects (i.e., up to 100 mg) is not toxic to mammals, and dependence is very rare [14]. Renal metabolism dominates, the main metabolites detected in urine are 3-indoleacetic acid and 3-indoacetic acid, and the half-life in the body ranges from a few minutes to several hours depending on the route of administration [14].

Recently, ketamine has emerged as a new therapeutic option for drug-resistant depression, which until recently was associated by psychiatrists primarily as an anesthetic or as a component of the patient’s polytoxicomania. At the same time, research is being conducted into the use of another substance that is illegal in most countries, 3,4-methylenedioxymethamphetamine (MDMA), popularly known as ecstasy, in the treatment of drug-resistant post-traumatic stress disorder (PTSD). Research is well advanced, and it is possible that MDMA will be approved as a drug by the FDA by 2022 [17]. For these reasons, scientists worldwide are exploring other, previously known as illicit, drug substances. According to the Global Drug Survey 2020 report by an independent UK scientific organization that studies the impact of psychoactive substance use on mental health, 8 of the 20 most commonly used psychoactive substances in 2020 are in the psychedelics and dissociatives group: in the past 12 months, 21.0% of respondents have used LSD, psilocybin mushrooms were used by 16.1% of respondents and DMT by 4.8% of respondents. These data come from over 110,000 individuals from over 25 countries, mostly in Europe [18]. By comparison, the 2019 Global Drug Survey reports that in the past 12 months, 17.5% of respondents have used LSD, 14.8% of respondents have used psilocybin mushrooms, and 4.2% of respondents have used DMT [19]. Equally popular is the interest in treating depression with psychedelics. On 23 June 2021, the Google search engine returned 2,310,000 results for the query “psychedelic treatment for depression”, and the topic is covered by well-known media outlets such as the BBC and Daily Mail [20,21]. Due to the increasing popularity of psychedelics in society, it is important to conduct further multidirectional research on them, including not only their use in therapy but also broadly understood public health issues, which is why the authors decided to conduct a systematic review of these substances in depression treatment.

The article presents a systematic review of LSD, psilocybin and DMT clinical use in depression treatment. The authors also aimed to create a rationale for further studies on use of psychedelics in psychiatry.

## 2. Material and Methods

**Figure 1:**
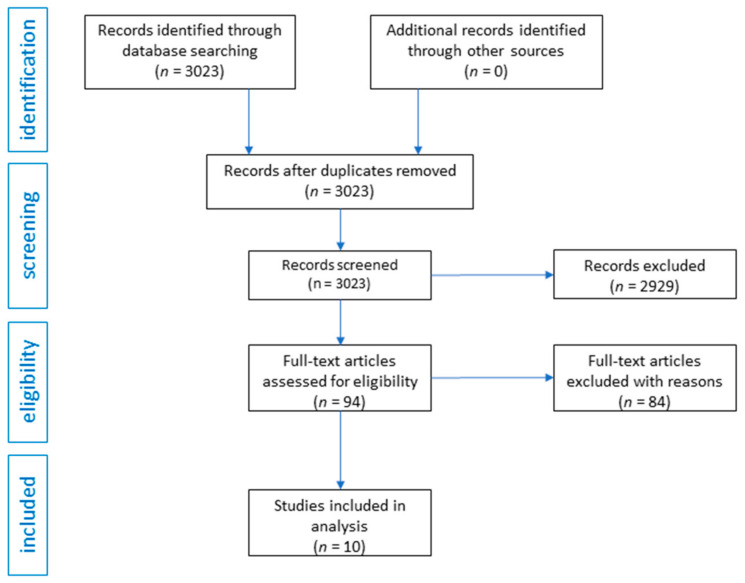
Flow diagram of systematic review of LSD, psilocybin and DMT in depression treatment.

Preferred Reporting Items for Systematic Reviews and Meta-Analyses (PRISMA) guidelines were followed while preparing this review. Two authors searched the PubMed and Cochrane Library databases independently to select those articles that would best reflect the assumptions of the article from the introduction. Chemical agents were combined with the terms “pharmacology”, “tolerance”, “depression”, “anxiety”, “meta-analysis” and “treatment” to find relevant articles (title/abstract). The authors included only original articles in English language that were related directly to LSD, psilocybin and DMT in depression treatment. The authors did not rule out articles due to the year of publication of the article. No other specific inclusion or exclusion criteria were used. The databases were searched in May–June 2021. The authors obtained 3023 records from the databases. After initial review, 2929 articles were excluded as they were review articles, editorials, commentaries, letters to editors, or they were not related to the topic of the review. In this way, 94 articles were obtained, of which 84 were excluded because they were studies that were not related directly to depression. The same two authors who searched the databases used the Effective Public Health Practice Project’s (EPHPP) Quality Assessment Tool for Quantitative Studies (QATQS) to assess risk of bias and study quality in quantitative studies. QATQS is a tool that allows evaluation of many different types of studies, including RCTs and non-RCTs. The tool contains different sections, each receiving a score of 1 (strong), 2 (moderate) or 3 (weak). QATQS contains questions such as selection bias, study design, confounders, blinding, data collection methods, withdrawals and drop-outs, intervention integrity and analyses. The final score is based on the number of weak ratings. One weak component results in a moderate rating, and two or more weak components result in a weak rating. Only studies without weak components have strong ratings [22,23]. The data extraction process focused on information about sample size, protocols of the studies and the outcomes. The authors conducted a narrative, qualitative summary. The flow diagram of the analysis is presented in Figure 1.

## 3. Results

### 3.1. Primary Outcome

All of the studies found had statistically significant results in lowering the intensity of depressive symptoms. A comprehensive report of the studies and their quality was presented in Table 1 and Table 2.

### 3.2. LSD

No clinical studies of LSD use in depression were found.

### 3.3. Psylocybin

Six studies were included in the analysis. The total number of participants included in the psilocybin studies ranged from 12 to 51, both male and female. Subjects were adults. In three studies the sample was composed of subjects with major depressive disorder [26,27,29], and in the other three studies the sample was composed of subjects with depression and anxiety in the course of life-threatening cancer [24,25,28]. A comprehensive report of findings is presented in Table 1.

### 3.4. DMT

Four studies were included in the analysis. The total number of participants included in the psilocybin studies ranged from 6 to 29, both male and female. Subjects were adults. In two studies the sample was composed of subjects with major depressive disorder [30,33], and in the other two studies the sample was composed of subjects with recurrent depression episodes [31,32]. A comprehensive report of findings is presented in Table 1.

## 4. Discussion

The use of psychedelics in depression treatment could be a safe treatment option, but further studies are necessary as there are some issues that researchers face.

The authors did not find any studies on LSD use in depression treatment even though the three substances that are the subject of this review share very similar mechanisms of action, which justifies conducting such studies.

Three out of six of the psilocybin studies included in this review were rated strong according to the Quality Assessment Tool for Quantitative Studies. Considering this and the fact that one other RCT study was rated moderate, psilocybin is the best documented substance in depression treatment from the three substances that are the subjects of this review, which makes psilocybin in a medical setting a very promising treatment option for patients with depression.

Only one out of four DMT studies included in this review was rated high according to the Quality Assessment Tool for Quantitative Studies. Three out of four of the DMT studies included were non-RCT studies, which do not allow to draw final conclusions on efficiency in depression treatment. No clinical studies of DMT use alone in depression treatment were found, as all of the included studies dosed ayahuasca, and it should be remembered that ayahuasca is administered with monoamine oxidase inhibitors, which may have an effect on mood themselves. It should also be remembered that ayahuasca contains other alkaloids, such as harmine or harmaline, and since it is a decoction of the plants themselves, it is difficult to estimate the dose of alkaloids consumed [34,35].

The use of psychedelic substances is not without risks. The pharmacology and mechanism of action of each substance is fairly well understood, and while LSD, psilocybin and DMT have relatively low health and life risks somatically, the risk of psychiatric complications must be considered. In addition to psychosis, another clinically significant complication that can occur after even a single ingestion of a psychedelic substance is Hallucinogen Persisting Perception Disorder or HPPD for short, classified as F16 in ICD-10 and 292.89 in DSM-V. This disorder manifests as chronic perceptual changes that can interfere with daily functioning and reduce quality of life and satisfaction. Duration is an individual matter, usually transient symptoms lasting from a few minutes to several months, although in extreme cases symptoms can last throughout life [36]. There are two types of HPPD: type 1, in which there is a brief recurrence of psychedelic effects in the form of a “flashback”, occurs in 1:20 people, and type 2, which occurs less frequently, in 1:50,000 and in which symptoms are chronic. Since the etiology and scientifically proven treatments remain unknown, HPPD should be considered a significant risk for patients undergoing treatment with psychedelics, according to the authors [37]. None of the studies included in the systematic review assessed occurrence of HPPD after clinical trials; therefore, further studies require assessing the psychological risks of psychedelics consumption.

## 5. Conclusions

By analyzing qualified studies, it can be concluded that psilocybin and DMT could be efficient and useful in depression treatment, but further studies and observations on bigger groups of patients are still required to assess safety. Considering social interest in psychedelics, studies for LSD use in depression treatment are urgent.

## Figures and Tables

**Table 1:** Summary of clinical studies evaluating the antidepressant effect of psilocybin (*n* = 6). The abbreviations are explained in the footer.

Authors	Year	Type of Study	Sample Size	Characteristic of Participants	Intervention	Results	Conclusions	QATQS Global Rating
Grob et al. [24]	2011	RCT	12	Subjects with depression and anxiety and advanced-stage cancer	Psylocybin in two sessions in several weeks interval (0.2 mg/kg) with 250 mg of niacin as a placebo As an efficacy measure BDI, STAI-S, STAI-T, Profile of Mood States (POMS) were used	BDI score were reduced at 6 months after treatment, STAI-T score reduction was observed at 1 and 3 months after treatment	Use of psilocybin combined with psychotherapy may provide an alternative treatment especially in the conditions with minimal response to conventional therapies, which needs to be investigated further in RCTs	1
Griffiths et al. [25]	2016	RCT	51	Subjects with depression or/and anxiety associated with life-threatening cancer	Psilocybin 22 or 30 mg/70 kg (high-dose) or placebo 1 or 3 mg/70 kg (low-dose) administered in controlled conditions in two sessions in 5 weeks interval. The effects were measured in GRID-HAM-D-17 scale and HAM-A assessed with the SIGH-A	Participants who get the high dose of psilocybin showed more significantly clinical response and symptom remission in GRID-HAM-D-17 and in HAM-A scale comparing to those patients who got low-dose therapy, those effects were sustained 6 months after treatment	Psilocybin decreases depressed mood as well as anxiety and also increase the quality of life in patients with a life-threatening cancer, the more various population of patients should be examined to evaluate the generality of psilocybin treatment	1
Carhart-Harris et al. [26]	2016	Non-RCT (open-label trial)	12	Subjects with treatment-resistant major depressive disorder (MDD)	Psylocybin 10 mg and 25 mg in two sessions with 7 days interval. Effects were assessed with QIDS-SR, Beck Depression Inventory (BDI), STAI, Snaith Hamilton Pleasure Scale (SHAPS), HAM-D, Montgomery-Asberg Depression Rating Scale and Global Assessment of Functioning (GAF)	BDI scores were reduced at 1 week, 3 and 6 months after treatment, STAI and SHAPS scores were reduced 1 week and 3 months after treatment, HAM-D and MADRS scores were reduced 1 week after treatment	Psilocybin is in need for further investigations in double-blind RCT as it seems to be effective in fighting drug-resistant MDD	3
Carhart-Harris et al. [27]	2018	Follow-up, Non-RCT (open-label trial)	20	Subjects with treatment-resistant major depressive disorder (MDD)	Psylocybin 10 mg and 25 mg in two sessions with 7 days interval. Effects were assessed with QIDS-SR (mainly) Beck Depression Inventory (BDI), STAI, Snaith Hamilton Pleasure Scale (SHAPS), HAM-D and Global Assessment of Functioning (GAF)	In 19 patients who completed all assesment time points, QIDS-SR16 scores were significantly reduced, BDI and STAI scores were reduced at 1 week, 3 and 6 months after treatment (*p* < 0.001), SHAPS scores were reduced at 1 week and 3 months after treatment (*p* < 0.001) and HAM-D and GAF scores were reduced 1 week after treatment (*p* < 0.001). No serious side-effects were observed during the treatment	Psilocybin is a promising tool in fighting unresponsive MDD and needs further investigations in double-blind RCT	3
Ross et al. [28]	2016	RCT	29	Subjects with depression and anxiety in life-threatening cancer	Psylocybin in two sessions (0.3 mg/kg) with a 7 days interval combined with psychotherapy and niacin (250 mg) as placebo. Efficacy was measured via STAI-T and STAI-S, HADS-A, HADS-D, HADS-T, BDI	Significant differences between study and control group, reductions on STAI-T, STAI-S, HADS-A( 58% vs. 14%), HADS-T, HADS-D and BDI (83% vs. 14%) in 1 day, 2, 6, and 7 weeks after first psylocybine session	In combination with psychotherapy in life-threatening illness psilocybin contributes to quick and sustained anti-depressant and anxiolytic effects	2
Davis et al. [29]	2020	RCT	24	Subjects with major depressive disorder (MDD)	Psilocybin 1 session 20 mg/70 kg, 2 session 30 mg/70 kg with supportive psychotherapy. Effects were evaluated in Hamilton Rating Scale for Depression (HAM-D) and in the Quick Inventory of Depressive Symptomatology-Self-Report (QIDS-SR)	After the session with psilocybin 71% of patients in 1 week and in 4 weeks showed more than 50% reduction in GRID-HAM score, 58% of participants in 1 week and 54% of participants in 4 weeks met the criteria of remission of depression; in QIDS-SR scale after psilocybin session the rapid, large decrease in mean depression score were observed which was remained 4 weeks after the treatment	Sessions with psilocybin-assisted therapy demonstrated large and sustained antidepressant effects among patients with MDD, however still further placebo-controlled studies are needed	1

QATQS—Quality Assessment Tool for Quantitative Studies, RCT—randomized controlled trial, Non-RCT—non- randomized controlled trial, MDD—major depressive disorder, MADRS—Montgomery-Åsberg Depression Rating Scale, HAM-D—Hamilton Rating Scale for Depression, HAM-D—Hamilton Rating Scale for Anxiety, QIDS-SR Quick Inventory of Depressive Symptomatology-Self-Report, BDI—Beck’s Depression Inventory, STAI-S—State-Trait Anxiety Inventory State, STAI-T—State-Trait Anxiety Inventory Trait, SHAPS—Snaith Hamilton Pleasure Scale, GAF—Global Assessment of Functioning, POMS—Profile of Mood States, HADS-A—Hospital Depression and Anxiety Scale-Anxiety, HADS-D—Hospital Depression and Anxiety Scale-Depression, HADS-T—Hospital Anxiety and Depression Scale-Total, SIGH-A—Structured Interview Guide for the Hamilton Anxiety Scale.

**Table 2:** Summary of clinical studies evaluating the antidepressant effect of DMT (*n* = 4). The abbreviations are explained in the footer.

Authors	Year	Type of Study	Sample Size	Characteristic of Participants	Intervention	Results	Conclusions	QATQS Global Rating
Palhano-Fontes et al. [30]	2019	RCT	29	Subjects with treatment-resistant major depressive disorder (MDD)	Patients received a single dose of either ayahuasca or placebo. Effects in depression severity were assessed with the Montgomery-Åsberg Depression Rating Scale (MADRS) and the Hamilton Depression Rating scale at baseline, and at 1 (D1), 2 (D2), and 7 (D7) days after dosing	Significant antidepressant effects of ayahuasca when compared with placebo at all-time points. MADRS scores were significantly lower in the ayahuasca group compared with placebo at D1 and D2 and at D7. Response rates were high for both groups at D1 and D2, and significantly higher in the ayahuasca group at D7 (64% vs. 27%). Remission rate showed a trend toward significance at D7 (36% vs. 7%)	This study brings new evidence supporting the safety and therapeutic value of ayahuasca, dosed within an appropriate setting, to help treat depression	1
Osório et al. [31]	2015	Non-RCT (open-label trial)	6	Subjects with current depressive episode	Patients received 120–200 mL of ayahuasca. Effects in depression severity were assessed with measured on the Hamilton Rating Scale for Depression (HAM-D), the Montgomery-Åsberg Depression Rating Scale (MADRS), and the Anxious-Depression subscale of the Brief Psychiatric Rating Scale (BPRS)	Statistically significant reductions of up to 82% in depressive scores were observed between baseline and 1, 7, and 21 days after ayahuasca administration	These results suggest that ayahuasca has fast-acting anxiolytic and antidepressant effects in patients with a depressive disorder	3
Sanches et al. [32]	2016	Non-RCT (open-label trial)	17	Subjects with recurrent depression episode	Patients receive ayahuasca (2.2 mL/kg) and were evaluated with the Hamilton Rating Scale for Depression, the Montgomery-Åsberg Depression Rating Scale, the Brief Psychiatric Rating Scale, the Young Mania Rating Scale, and the Clinician Administered Dissociative States Scale during acute ayahuasca effects and 1, 7, 14, and 21 days after drug intake	Increased psychoactivity (Clinician Administered Dissociative States Scale) and significant score decreases in depression-related scales (Hamilton Rating Scale for Depression, Montgomery-Åsberg Depression Rating Scale, Brief Psychiatric Rating Scale) from 80 min to day 21	Results suggest that ayahuasca may have fast-acting and sustained antidepressive properties. These results should be replicated in randomized, double-blind, placebo-controlled trial.	3
Zeifman et al. [33]	2020	Non-RCT (open-label trial)	17	Subjects with major depressive disorder (MDD)	Patient received single dose of ayahuasca and were evaulated with The Montgomery-Åsberg Depression Rating Scale	Among individuals with suicidality at baseline (*n* = 15), there were significant acute (i.e., 40, 80, 140, and 180 min after administration) and post-acute (1, 7, 14, and 21 days after administration) decreases in suicidality following administration of ayahuasca	Ayahuasca could possibly lead to rapid and sustained reductions in suicidality among individuals with MDD Randomized, double-blind studies with larger sample sizes are needed to confirm this early finding	3

QATQS—Quality Assessment Tool for Quantitative Studies, RCT—randomized controlled trial, Non-RCT—non- randomized controlled trial, MDD—major depressive disorder, BPRS—Brief Psychiatric Rating Scale, MADRS—Montgomery-Åsberg Depression Rating Scale, HAM-D—Hamilton Rating Scale for Depression.

## Data Availability

Data sharing not applicable.
